# Psychosocial characteristics of primary care-seeking long-distance truck drivers in Kenya and associations with HIV testing

**DOI:** 10.2989/16085906.2018.1449760

**Published:** 2018-04-24

**Authors:** Matthew L Romo, Gavin George, Joanne E Mantell, Eva Mwai, Eston Nyaga, Jacob O Odhiambo, Kaymarlin Govender, Elizabeth A Kelvin

**Affiliations:** 1 Department of Epidemiology and Biostatistics & Institute for Implementation Science in Population Health, CUNY Graduate School of Public Health and Health Policy, City University of New York, New York, USA; 2 Health Economics and HIV and AIDS Research Division, University of KwaZulu-Natal, Durban, South Africa; 3 North Star Alliance, Nairobi, Kenya; 4 HIV Center for Clinical and Behavioral Studies, Department of Psychiatry, Division of Gender, Sexuality, and Health, New York State Psychiatric Institute & Columbia University Medical Center, New York, USA

**Keywords:** attitude to health, masculinity, risk-taking, self-concept, self-efficacy, social stigma

## Abstract

The 90-90-90 strategy from the Joint United Nations Programme on HIV/AIDS (UNAIDS) to end the AIDS epidemic by 2020 includes, as its first goal, to have 90% of all people living with HIV to know their status. Achieving this goal will depend on effectively reaching high risk populations, which include mobile populations such as truck drivers. This study aimed to characterise a sample of 305 truck drivers recruited from 2 roadside wellness clinics in Kenya in terms of anticipated HIV stigma, self-efficacy, fatalism, gender equity, sensation seeking, and self-esteem, and then determine the association of these psychosocial characteristics with HIV testing behaviour. Greater general self-efficacy was associated with higher income and more years working as a truck driver. Greater fatalism was associated with non-Christian religion, being married, and having a lower income. Greater gender equity was associated with completing high school, being married, and having higher income. Greater sensation seeking was associated with lower income and fewer years employed as a truck driver. In multivariable logistic regression adjusted for demographic variables, anticipated HIV stigma was negatively associated with having ever tested for HIV (adjusted odds ratio [aOR] = 0.79; 95% confidence interval [CI] = 0.63–0.98; *p* = 0.034) and self-esteem was positively associated with testing (aOR = 1.06; 95% CI = 1.00–1.12; *p* = 0.038). Associations with HIV testing behaviour were not significant for self-efficacy, fatalism, gender equity, or sensation seeking. Public health interventions aiming to reduce anticipated stigma and increase self-esteem may potentially increase the uptake of HIV testing among truck drivers. Further research is needed to better understand the influence of these psychosocial characteristics on HIV testing.

## Introduction

Several studies have indicated that long-distance truck drivers are disproportionally affected by the HIV epidemic in sub-Saharan Africa (Azuonwu, Erhabor, & Frank-Peterside, 2011; Botão et al., 2016; Delany-Moretlwe et al., 2014; Diallo, Alary, Rashed, & Barry, 2011; Mosoko, Macauley, Zoungkanyi, Bella, & Koulla-Shiro, 2009). Truck drivers are considered to be at increased risk for the aquistion of HIV because their mobility and extended time away from their main partners make them prone to engaging in risky sexual behaviours, including multiple and concurrent sexual partners, use of commercial sex services, and inconsistent condom use (Delany-Moretlwe et al, 2014; Lagarde et al., 2003; Morris & Ferguson, 2007). Mobility reduces truck drivers’ acess to HIV services and many of these drivers remain untested and unaware of their HIV-postive status. In Kenya, the focus of our study, a prospective cohort study conducted more than 20 years ago at a trucking company in Mombasa indicated HIV prevalence was at 17.8%, with significantly higher hazard ratios for sero-conversion for truck drivers and their assistants as compared with stationary staff (Rakwar et al., 1999). At a national level in Kenya, HIV prevalence in 2012 was estimated at 5.6% among adults 15–64 years of age (4.4% among men and 6.9% among women) (NASCOP, 2014).

Although HIV testing uptake in Kenya has increased nationwide, gender disparities remain, with men consistently testing less frequently than women (NASCOP, 2014). Considering that truck driving is a male-dominated profession in Kenya, we would expect HIV testing rates to be lower in this population than in the general population. However, data on testing in this population are scant. According to the North Star Alliance, which runs roadside wellness clinics in sub-Saharan Africa, including 8 clinics in Kenya, among the 143 608 clinic visits in 2015 by men, about two-thirds of whom were truck drivers, only 20% included HIV testing (North Star Alliance, 2015a). Despite the sparse data on HIV prevalence and testing among truck drivers in Kenya, the government recognises this population as being particularly susceptible to HIV infection and in need for targeted HIV testing services (NASCOP, 2015). The North Star Alliance is currently running an intervention to incentivise HIV testing and utilisation of other preventive health services in their STAR DRIVER programme in the country (North Star Alliance, 2015b). In addition, the Kenya Long Distance Truck Drivers’ Union has been supported by the Swedish Workplace HIV/AIDS Programme and the International Labour Organization to provide HIV testing and education to trucker drivers (Swedish Workplace HIV/AIDS Programme, 2015).

Given that barriers to HIV testing are multidimensional, several are psychosocial in nature and can have an impact on an individual’s perceptions about testing (Evangeli, Pady, & Wroe, 2016; Musheke et al, 2013). Potential psychosocial barriers to HIV testing include anticipated HIV stigma (e.g., concern that knowing one’s HIV status might lead to rejection by family, friends, and/or colleagues) (Pulerwitz, Michaelis, Lippman, Chinaglia, & Diaz, 2008); self-efficacy (e.g., lack of self-belief in ability to test for HIV and reduce risk behaviour) (Berendes & Rimal, 2011); fatalism (e.g., susceptibility to HIV is determined by fate, so there is not much benefit to testing) (Obermeyer & Osborn, 2007; Ojikutu, Nnaji, Sithole-Berk, Bogart, & Gona, 2014); gender norms (e.g., HIV testing may be perceived as incongruous with traditional male gender roles because seeking health care may be a sign of less “manliness”) (DiCarlo et al, 2014; Fleming, Colvin, Peacock, & Dworkin, 2016); sensation seeking (e.g., predilection for high risk behaviour may lead to lower engagement in health-protective behaviour, such as HIV testing) (Kalichman, Simbayi, Jooste, Cain, & Cherry, 2006; Kalichman, Simbayi, Jooste, Vermaak, & Cain, 2008); and self-esteem (e.g., poor self-opinion leads to worse self-care and less HIV testing) (Stein & Nyamathi, 2000).

Limited data exist on the psychosocial characteristics of truck drivers in sub-Saharan Africa in general and how these characteristics might influence HIV testing behaviour. Outside Africa, a study of truck drivers in Brazil reported that anticipated HIV stigma was negatively associated with having ever tested for HIV (Pulerwitz et al., 2008). Low self-efficacy has been associated with lower HIV testing rates in general population samples in sub-Saharan African settings outside Kenya (Berendes & Rimal, 2011). In sub-Saharan Africa, truck drivers have been described as having fatalistic attitudes and lack control over becoming infected with HIV (IRIN, 2013; Progressio, 2013). While no published studies exist on the association of fatalism with HIV testing among truck drivers, fatalism was found to be negatively associated with HIV testing in a sample of black immigrants in the United States (Ojikutu et al., 2014). Research on gender norms and equity has been mostly qualitative. For example, studies that have included men and women suggest that hegemonic masculine norms might explain lower HIV testing rates among men in in South Africa and Lesotho (DiCarlo et al., 2014; Fleming et al., 2016). Sensation seeking is associated with sexual risk behavior (Kalichman et al., 2006; Kalichman et al., 2008), which might influence the decision to test for HIV. Self-esteem has been reported to be positively associated with HIV testing in a high risk sample in the United States and was mediated through positive coping (Stein & Nyamathi, 2000).

Given the paucity of data on the relationship between psychosocial variables and HIV testing behaviours, this study aimed to document the prevalence of various psychosocial variables (i.e., anticipated HIV stigma, self-efficacy, fatalism, gender equity, sensation seeking and self-esteem) in a sample of truck drivers in Kenya and determine whether these psychosocial variables were associated with HIV testing behaviour.

## Methods

### Study procedures

Data were collected from a randomised controlled trial evaluating whether offering HIV testing options (i.e., provider-administered blood-based finger prick rapid HIV testing, self-administered oral rapid HIV testing (OraQuick In-Home HIV Test) in the clinic with supervision or a self-test kit for home use) versus the standard of care (i.e., provider-administered blood-based finger prick testing) could increase HIV testing among truck drivers in Kenya (Kelvin et al., 2017a; Kelvin et al., 2018). The study procedures were reviewed and approved by the City University of New York (CUNY) Institutional Review Board, the Ethics Committees of the Kenya Medical Research Institute (KEMRI) and the Biomedical Research Ethics Committee (BREC) at the University of KwaZulu-Natal in South Africa.

Participants were recruited from two North Star Alliance roadside wellness clinics in Kenya located along major transportation corridors in Nakuru county. Male truck drivers who visited the two clinics between October and December 2015 for services other than HIV treatment were informed about the study and, if interested, underwent eligibility screening. Eligibility criteria were: (1) ≥18 years of age; (2) male; (3) employed as a truck driver; (4) primary residence in Kenya; (5) able to speak English or Kiswahili; (6) self-reported HIV-negative or unknown HIV status; (7) able to sign their name on the consent form; and (8) able to receive payment for participation via M-Pesa, a mobile phone-based money transfer system widely used in Kenya. Female truck drivers were not included as the proportion of truck drivers at North Star Alliance clinics in Kenya who are female is estimated to be <1%. Overall, 319 male truck drivers were screened for eligibility and of the 14 excluded, reasons were: refusal to participate in the study (*n* = 6); HIV-positive (*n* = 5); not being a resident of Kenya (*n* = 1); unable to sign name (*n* = 1); and unwilling to use M-Pesa (*n* = 1). A total of 305 male truck drivers were enrolled and randomised to be offered either the provider-administered finger prick testing (standard of care arm, *n* = 155) or offered both standard provider-administered testing and oral self-testing (choice arm, *n* = 150), stratified by clinic (clinic 1, *n* = 144; clinic 2, *n* = 161).

Fieldworkers administered a baseline survey which included questions about demographic characteristics, HIV testing history, and several psychosocial scales. Following the baseline interview, participants were offered HIV testing, with the options dependent on the randomisation arm. After undergoing or refusing HIV testing, appointments were made for a follow-up interview at 6 months. Participants in the choice arm were told that they could pick up an oral self-testing kit at any of the eight North Star Alliance clinics in Kenya during the follow-up period and received reminders via text message 3 months after the baseline interview. Those in the standard of care arm were also sent a text message 3 months after the baseline interview reminding them that HIV testing (but not self-testing) was available at North Star Alliance clinics, which is the standard practice at the North Star Alliance.

At the 6-month follow-up interview, research staff administered the self-esteem scale and asked about HIV testing in the past 6 months. Questionnaires were administered in English or Kiswahili, depending on the participant’s preference. Participants were compensated 270 Kenya shillings (KES) for the baseline interview, KES270 for the post-test interview, and KES360 for the follow-up interview. At the time of the study, KES100 was equivalent to US$1.

To maintain high data quality, research staff periodically downloaded the data, identified responses that were missing without a reason, such as participant refusal, or were illogical, and queried staff at the North Star Alliance clinic who would then compare the responses with the hard copy files or consult the fieldworkers. Any responses that could not be clarified were classified as missing. In addition, all responses to questions that participants refused to answer or did not provide due to loss to follow-up were also classified as missing.

### Measures

#### HIV testing outcomes

Four measures of HIV testing behaviour were examined: (1) self-report of having ever tested for HIV; (2) self-report of testing in the past 6 months, based on response to the question of how long it had been since their last HIV test. Those who had never tested were coded as not having tested in the past 6 months; (3) tested at baseline, which was based on whether the participant accepted HIV testing when it was offered following the baseline interview; and (4) self-report at the follow-up interview of testing during the 6 months following the baseline interview.

#### Demographics

Age was categorised as <40 and ≥40 years for bivariate analyses and was retained as a continuous variable for regression. Religion was dichotomised as Christian (i.e., Protestant or Catholic) or non-Christian (i.e., Muslim, Hindu, traditional African and no religion). Educational attainment was categorised as having matriculated from high school versus lower than high school matriculation (i.e., no education, some primary school, completed primary school only or some high school). Marital status was categorised as currently married (i.e., legal or common-law) versus unmarried (i.e., divorced/separated, widowed or single). Average monthly income was based on a self-reported estimate or if unable or unwilling to specify an exact amount, based on income range categories in KES: <8 000, 8 000–16 000, 16 001–24 000, 24 001–50 000, or >50 001. Income was collapsed into 2 categories for analysis: ≤24 000 and >24 000. Participants were asked how many months and years they had worked as a truck driver. Responses were categorised as <10 years and ≥10 years for bivariate analyses and used as a continuous variable for regression.

#### Psychosocial scales

Six psychosocial scales were examined, with summary scores calculated as described in the following section while allowing up to 20% of the items (1 or 2 items, depending on the scale) to be left unanswered for each scale, as done in a previous study (Kelvin et al., 2017b).

1. Anticipated HIV stigma. A nine-item scale, previously used in Botswana (Weiser et al., 2006), presented yes/no statements about possible stigma-related scenarios if the participant were to test positive for HIV and others found out about their status. The scale had a possible score range of 0–9, with higher scores indicating more anticipated stigma. The scale demonstrated good reliability (Cronbach’s α = 0.80).
2. General self-efficacy. A 10-item scale (Schwarzer & Jerusalem, 1995), with previous multicultural validation (Luszczynska, Scholz, & Schwarzer, 2005), presented statements related to belief in one’s confidence to cope with a broad range of stressful or challenging demands. The response categories ranged from “not at all true” to “exactly true”, with a possible score range of 10–40; higher scores indicated greater self-efficacy. The scale demonstrated excellent reliability (Cronbach’s α = 0.89).
3. Fatalism. A 20-item scale (Shen, Condit, & Wright, 2009) elicited responses to a series of fatalistic statements mostly related to health, with a 5-point Likert scale ranging from “strongly disagree” to “strongly agree”. The scale had a possible score range of 20–100, with higher scores indicating more fatalistic views. The scale demonstrated excellent reliability (Cronbach’s α = 0.93).
4. Gender equity. A 24-item scale (Pulerwitz & Barker, 2008), widely used in sub-Saharan Africa (Shattuck et al., 2013), consisted of statements related to relationships between men and women, with responses consisting of “agree”, “partially agree”, and “do not agree”. The scale had a possible score range of 24–72, with higher scores indicating more gender-equitable attitudes. The scale demonstrated excellent reliability (Cronbach’s α = 0.88).
5. Sensation seeking. A five-item scale (Kalichman et al., 1994), previously adapted for use in South Africa (Kalichman et al., 2006; Kalichman et al., 2008), contained statements about self-perceived propensity for pleasure associated with risk taking. Response categories on this four-point Likert scale ranged from “not at all like me” to “very much like me”. The first item from the scale was dropped to improve internal consistency, so the scale had a possible score range of 4–16 with higher scores indicating greater sensation seeking. The scale demonstrated acceptable reliability (Cronbach’s α = 0.73).
6. Self-esteem. A 10-item scale (Rosenberg, 1965), widely used globally, including in sub-Saharan Africa (Schmitt & Allik, 2005), consisted of statements about perceived self-esteem, with a 4-point Likert scale ranging from “strongly agree” to “strongly disagree”. The scale had a possible score range of 0–30, with a higher score indicating greater self-esteem. This scale was only administered at follow-up, resulting in 21 fewer participants from the baseline study population who did not have results for this scale, since they did not present for their follow-up interview. The scale demonstrated excellent reliability (Cronbach’s α = 0.88).

### Statistical analysis

First, the overall sample was described in terms of demographics, HIV testing, and scores on the psychosocial scales. Second, the score distributions for each scale were compared by demographic and HIV testing behaviour variables using the Mann–Whitney *U*-test, as most scale scores were not normally distributed. Third, correlations among the scales were determined. Spearman correlation coefficients were calculated as they are robust to outliers, which were present for some of the scales. Finally, unadjusted crude and adjusted multivariable logistic regression analyses were run to determine the association of each psychosocial scale with each HIV testing behaviour outcome. All multivariable models were adjusted for age, religion, educational level, marital status, monthly income, years worked as a truck driver, and clinic. The models for testing at baseline and testing at follow-up were also adjusted for randomisation assignment. The multivariable models were adjusted for these demographic variables in an effort to see if associations held from the unadjusted crude models. Listwise deletion was used so that any participant with missing data was dropped from the models. The odds ratios (ORs) from these models can be interpreted as the odds of the HIV testing outcome per one-point increase in the scale score. The significance level was set at a two-sided α of 0.05. All analyses were conducted in SAS 9.4 (SAS Institute Inc., Cary, NC).

## Results

### Description of sample

Of the 305 male truck drivers included in this study, most were younger than 40 years old (66.9%), Christian (77.9%), had not completed high school (64.3%), married (83.1%), had a monthly income of more than KES24 000 (72.2%), and had worked as a truck driver for less than 10 years (64.3%). Most participants (91.8%) reported having previously tested for HIV and 48.2% reported testing in the past 6 months. When offered HIV testing following the baseline interview, 80.0% accepted and were tested. At the 6-month follow-up, 56.0% of participants reported testing since the baseline interview (Table 1).

### Bivariate analyses of psychosocial scales with demographic and HIV testing behaviour variables

Anticipated HIV stigma scores were significantly higher among participants who reported they had never tested for HIV (mean score 1.6 vs 0.6 among those who had previously tested; *p* = 0.001). Self-efficacy scores were significantly higher among participants earning more than KES24 000 monthly (mean score 37.0 vs 33.9 for those earning ≤KES24 000; *p* = 0.001), and participants who had worked for at least 10 years as a truck driver (mean score 37.8 vs 35.4 for those who had worked <10 years; *p* = 0.001). Fatalism scores were significantly higher among participants identifying as non-Christian (mean score 55.2 vs 44.3 for Christian; *p* = 0.001); those who were married (mean score 48.1 vs 41.0 for those unmarried; *p* = 0.025); those who had a monthly income of ≤KES24 000 (mean score 53.0 vs 45.6 for those making >24 000; *p* = 0.005); and those with <10 years working as a truck driver (mean score 48.4 vs 44.1 for ≥10 years; *p* = 0.044). Gender equity scores were significantly higher among participants who had completed high school (mean score 61.3 vs 58.3 for those with less education; *p* = 0.005); were married (mean score 62.9 vs 58.6 for those who were unmarried; *p* = 0.002); were earning more than KES24 000 per month (mean score 60.1 vs 57.4 for those earning ≤KES24 000; *p* = 0.008); and participants who had refused testing at baseline (mean score 61.6 vs 58.8 for those who accepted testing; *p* = 0.030). Sensation seeking scores were significantly higher for participants with a monthly income of ≤KES24 000 (mean score 6.7 vs 5.4 for those making >KES24 000; *p* = 0.001) and among those who had worked as truck drivers for <10 years (mean score 6.2 vs 4.9 for those who had worked ≥10 years; *p* = 0.001). Self-esteem scale scores were significantly higher for participants who had previously tested for HIV (mean score 20.9 vs 18.1 for those who had never tested; *p* = 0.024) (Table 1).

### Correlations among psychosocial scales

There was low correlation among the scales, with the exception of a moderate negative correlation between sensation seeking and self-efficacy (*r* = −0.50; *p* = 0.001) and between gender equity and fatalism (*r* = −0.59; *p* = 0.001) (Table 2).

## Logistic regression results for HIV testing behaviours

In the crude models, anticipated HIV stigma (crude odds ratio [cOR] = 0.73; *p* = 0.002) and self-esteem (cOR = 1.11; *p* = 0.029) were significantly associated with having ever tested for HIV (Table 3). The association of fatalism and having ever tested approached statistical significance (cOR = 0.98; *p* = 0.060). In the multivariable (i.e., adjusted) models, only anticipated HIV stigma (adjusted odds ratio [aOR] = 0.79; *p* = 0.034) was significantly associated with lower odds of having ever tested for HIV. In crude models and multivariable models, none of the psychosocial scales were significantly associated with having tested in the past 6 months. In crude models, gender equity (cOR = 0.96; *p* = 0.032) and sensation seeking (cOR = 1.14; *p* = 0.049) were significantly associated with testing at baseline. However, none of the psychosocial scales had significant associations in the multivariable models. In crude models, none of the psychosocial scales were significantly associated with having tested during follow-up, but in the multivariate models, self-esteem was significantly associated with having testing during follow-up (aOR = 1.06; *p* = 0.036) (Table 3).

## Discussion

Truck drivers in this study scored in the very low range on the anticipated HIV stigma scale (mean 0.6 out of a possible range of 0–9); in the high range on the self-efficacy scale (mean 36.3 out of a possible range of 10–40); in the low-mid range on the fatalism scale (mean 46.9 out of a possible range of 20–100), in the mid-high range on the gender equity scale (mean 59.4 out of a possible range of 24–72); in the low-mid range on the sensation seeking scale (mean 5.7 out of a possible range of 4–16); and in the mid-high range on the self-esteem scale (mean 20.6 out of a possible range of 0–30). Truck drivers in this study had lower mean anticipated HIV stigma scores than a general population sample in Botswana (approximately 1.9) (Weiser et al., 2006) and lower mean anticipated stigma scores than a sample of pregnant women in rural Kenya (approximately 2.5) (Turan et al., 2011). This lower anticipated stigma appears consistent with the fact that most of the sample had previously tested for HIV, thus overcoming any initial barriers to testing. Although there are no published studies among African populations with the fatalism scale used in this study, the mean score in this study’s sample was similar to that of adults living in the rural United States with cardiovascular disease risk factors (43.7) (Mudd-Martin et al., 2015). Thus, the description of truck drivers in Africa as being very fatalistic (IRIN, 2013; Progressio, 2013) may be an overstatement. Participants had a slightly higher mean score on the gender equity scale than samples of adult men from Tanzania (approximately 53.0) and Ghana (approximately 47.0) (Shattuck et al., 2013). The mean self-efficacy, sensation seeking, and self-esteem scores among study participants were fairly consistent with scores from other general adult populations in sub-Saharan Africa (Kalichman et al., 2006; Schmitt & Allik, 2005; Williams, Wissing, Rothmann, & Temane, 2010).

We hypothesised that certain psychosocial characteristics would be associated with HIV testing behaviour. Although the results indicate that anticipated HIV stigma and self-esteem may be related to some HIV testing outcomes, the inconsistency of these associations across HIV testing outcomes, as well as lack of association for other psychosocial scales, especially fatalism and self-efficacy, did not fully support this. This lack of consistency across outcomes might also indicate that some of the HIV testing outcomes have different associations with psychosocial characteristics.

The association between anticipated HIV stigma and having never tested is consistent with a study conducted among truck drivers in Brazil (Pulerwitz et al., 2008) and other studies of non-truck driver populations done in sub-Saharan Africa (Kalichman & Simbayi, 2003; Kelly, Weiser, & Tsai, 2016; Mall, Middelkoop, Mark, Wood, & Bekker, 2013; Young et al., 2010) reporting that HIV/AIDS stigma is negatively associated with testing. Self-esteem was significantly associated only with having tested at follow-up. In the literature there appears to be divergent results for the association between self-esteem and HIV testing, as a study in the United States indicated that self-esteem was associated with testing (Stein & Nyamathi, 2000), but a study in Uganda found a null association between self-esteem and having ever tested for HIV among adolescents (Hampanda, Ybarra, & Bull, 2014). The relationship between self-esteem and HIV testing warrants further exploration.

Fatalism was not significantly associated with HIV testing behaviour, which is contrary to a substantial body of literature implicating unrealistic perceptions about HIV risk and transmission as a barrier to testing (Obermeyer & Osborn, 2007). We had predicted that fatalism would decrease the perception that HIV testing is useful and thus would be a barrier to testing, but the data did not support this. The fatalism scale in this study was general and not specific to HIV, which might explain the lack of association. Other unexpected findings were the lack of association of self-efficacy, gender equity, and sensation seeking with HIV testing. However, all study participants were recruited from clinics. Some of these psychosocial characteristics could affect the probability of seeking health care in general (e.g., self-efficacy) and that once the individual is linked to services, whether for HIV testing or other services, the probability of HIV testing may be attributed to other factors, such as the provider’s communication skills or the available amount of time the individual has to spend at the clinic.

There are several limitations to consider when interpreting these data. First, we conducted several statistical models and therefore some of the statistical associations may have been spurious. Second, the sample size was fairly small and the study was not powered specifically to look at these associations, so there may not have been sufficient statistical power to detect some associations. Third, most outcomes, except testing in the clinic at baseline, were based on self-report and there may have been some recall and social desirability bias reflected in the results. Fourth, the multivariable models were only adjusted for demographic characteristics, so confounding may have biased some of the associations. Finally, the findings from this study may not be generalisable to all truck drivers in Kenya or in other countries, particularly since the study population was recruited from a clinic.

## Conclusions

This study’s findings are generally consistent with those in the literature regarding the impact of anticipated HIV stigma and therefore programmes that aim to increase HIV testing among truck drivers need to consider this. One promising avenue of intervention may be through complementary inclusion of peers in the HIV testing process and/or environment (WHO, 2015). Health education from HIV-positive peers is likely to reduce anticipated HIV stigma through debunking some misunderstandings about living with HIV. Because self-testing is currently being implemented in several sub-Saharan African countries, including Kenya (UNAIDS, 2017), and has the potential to increase HIV testing uptake (Heard & Brown, 2016), expanding this service among truck drivers in Kenya (Kelvin et al., 2018) is seen as the next logical step in HIV programming. The results of this study can serve as a baseline to inform how we plan these proposed interventions. Further research on HIV testing behaviours among truck drivers in Africa should also focus on non-clinic-based samples, and include larger samples to rule out insufficient statistical power as an explanation for some of the null findings in this study.

## Figures and Tables

**Table 1:** Mean scores on psychosocial scales and distribution by demographics and HIV testing behaviour

	Total	Anticipated HIV stigma *N* = 305	Self-efficacy *N* = 305	Fatalism *N* = 305	Gender equity *N* = 305	Sensation-seeking *N* = 305	Self-esteem *N* = 284
	*n* (%)	Mean (SD)	Mean (SD)	Mean (SD)	Mean (SD)	Mean (SD)	Mean (SD)
Possible score range	–	0 (low) to	10 (low) to	20 (low) to	24 (low) to	4 (low) to	0 (low) to
		9 (high)	40 (high)	100 (high)	72 (high)	16 (high)	30 (high)
Overall mean (SD) score	–	0.6 (1.4)	36.3 (4.9)	46.9 (20.7)	59.4 (8.9)	5.7 (2.5)	20.6 (5.5)
Overall median (IQR) score	–	0.0 (1.0)	38.0 (6.0)	48.0 (37.0)	59.0 (12.0)	4.0 (3.0)	19.0 (9.0)
Age	*N* = 305	*p* = 0.953	*p* = 0.485	*p* = 0.354	*p* = 0.106	*p* = 0.918	*p* = 0.131
<40 years	204 (66.9%)	0.7 (1.4)	36.4 (4.8)	46.3 (19.9)	60.1 (8.3)	5.7 (2.5)	20.4 (5.4)
≥40 years	101 (33.1%)	0.6 (1.4)	35.9 (5.2)	48.0 (22.2)	58.0 (10.0)	5.8 (2.7)	21.2 (5.8)
Religion	*N* = 299	*p* = 0.075	*p* = 0.253	*p* = 0.001	*p* = 0.027	*p* = 0.130	*p* = 0.582
Christian	233 (77.9%)	0.6 (1.3)	36.1 (5.1)	44.3 (19.3)	60.1 (8.3)	5.8 (2.6)	20.8 (5.7)
Non-Christian	66 (22.1%)	1.0 (1.8)	37.3 (3.6)	55.2 (23.6)	56.5 (10.7)	5.2 (2.2)	20.2 (5.2)
High school graduate	*N* = 305	*p* = 0.092	*p* = 0.764	*p* = 0.098	*p* = 0.004	*p* = 0.989	*p* = 0.977
Yes	109 (35.7%)	0.7 (1.2)	36.6 (4.6)	44.0 (16.9)	61.3 (7.9)	5.7 (2.5)	20.5 (5.0)
No	196 (64.3%)	0.6 (1.5)	36.1 (5.0)	48.4 (22.4)	58.3 (9.3)	5.7 (2.6)	20.7 (5.8)
Married	*N* = 302	*p* = 0.540	*p* = 0.112	*p* = 0.025	*p* = 0.002	*p* = 0.275	*p* = 0.618
Yes	251 (83.1%)	0.7 (1.5)	36.1 (5.0)	48.1 (20.8)	62.9 (8.0)	5.8 (2.6)	20.5 (5.5)
No	51 (16.9%)	0.5 (1.2)	37.1 (4.6)	41.0 (19.5)	58.6 (9.0)	5.3 (2.2)	21.1 (5.7)
Mean income per month	*N* = 288	*p* = 0.471	*p* = 0.001	*p* = 0.005	*p* = 0.008	*p* = 0.001	*p* = 0.868
≤24 000 KES	80 (27.8%)	0.8 (1.7)	33.9 (6.0)	53.0 (20.2)	57.4 (8.6)	6.7 (2.8)	20.3 (5.0)
>24,000 KES	208 (72.2%)	0.6 (1.3)	37.0 (4.2)	45.6 (20.2)	60.1 (8.8)	5.4 (2.4)	20.5 (5.5)
Number of years worked as a truck driver	*N* = 305	*p* = 0.835	*p* = 0.001	*p* = 0.044	*p* = 0.614	*p* = 0.001	*p* = 0.953
<10 years	196 (64.3%)	0.6 (1.3)	35.4 (5.4)	48.4 (19.4)	59.4 (8.6)	6.2 (2.8)	20.5 (5.3)
≥10 years	109 (35.7%)	0.7 (1.5)	37.8 (3.4)	44.1 (22.6)	59.4 (9.5)	4.9 (1.8)	20.8 (6.0)
Ever tested for HIV	*N* = 305	*p* = 0.001	*p* = 0.537	*p* = 0.056	*p* = 0.872	*p* = 0.561	*p* = 0.024
Yes	280 (91.8%)	0.6 (1.4)	36.2 (4.9)	46.2 (21.0)	59.4 (8.9)	5.8 (2.6)	20.9 (5.6)
No	25 (8.2%)	1.6 (1.7)	36.9 (4.3)	54.4 (14.7)	59.4 (9.1)	5.3 (2.0)	18.1 (4.5)
Tested for HIV in past 6 months	*N* = 305	*p* = 0.164	*p* = 0.811	*p* = 0.748	*p* = 0.171	*p* = 0.882	*p* = 0.160
Yes	147 (48.2%)	0.6 (1.4)	36.2 (4.8)	46.5 (19.5)	60.1 (8.2)	5.7 (2.5)	20.3 (5.3)
No	158 (51.8%)	0.7 (1.4)	36.3 (5.0)	47.2 (21.8)	58.7 (9.5)	5.8 (2.6)	21.0 (5.7)
Accepted HIV testing at baseline	*N* = 305	*p* = 0.112	*p* = 0.808	*p* = 0.947	*p* = 0.030	*p* = 0.091	*p* = 0.539
Yes	244 (80.0%)	0.6 (1.4)	36.1 (5.2)	46.9 (21.6)	58.8 (9.0)	5.9 (2.6)	20.8 (5.7)
No	61 (20.0%)	0.9 (1.5)	37.0 (3.5)	46.8 (16.8)	61.6 (8.6)	5.1 (2.1)	20.1 (5.0)
Tested during follow-up	*N* = 284	*p* = 0.178	*p* = 0.889	*p* = 0.611	*p* = 0.617	*p* = 0.414	*p* = 0.124
Yes	159 (56.0%)	0.7 (1.4)	36.7 (4.2)	45.7 (20.1)	59.4 (9.5)	5.4 (2.2)	21.0 (5.6)
No	125 (44.0%)	0.7 (1.5)	36.4 (5.2)	47.2 (21.8)	59.4 (8.7)	5.8 (2.6)	20.2 (5.5)

KES = Kenya shilling; IQR = interquartile range; SD = standard deviation

**Table 2:** Correlation matrix of psychosocial scales (Spearman correlation coefficients}

	Anticipated HIV stigma	Self-efficacy	Fatalism	Gender equity	Sensation-seeking	Self-esteem
Anticipated HIV stigma	*r* = 1.00					
Self-efficacy	*r* = −0.03	*r* = 1.00				
	*p* = 0.590					
Fatalism	*r* = 0.17	*r* = −0.41	*r* = 1.00			
	*p* = 0.003	*p* < 0.001				
Gender equity	*r* = −0.15	*r* = 0.33	*r* = −0.59	*r* = 1.00		
	*p* = 0.009	*p* < 0.001	*p* < 0.001			
Sensation-seeking	*r* = −0.003	*r* = −0.50	*r* = 0.36	*r* = −0.31	*r* = 1.00	
	*p* = 0.959	*p* = 0.001	*p* = 0.001	*p* = 0.001		
Self-esteem	*r* = −0.10	*r* = 0.17	*r* = −0.38	*r* = 0.12;	*r* = −0.03	*r* = 1.00
	*p* = 0.083	*p* = 0.003	*p* = 0.001	*p* = 0.052	*p* = 0.644	

**Table 3:** Logistic regression models for the association between psychosocial scale scores and HIV testing behaviour

	Ever tested for HIV		Tested for HIV in past 6 months		Accepted HIV testing at baseline		Tested for HIV during follow-up	
	Crude models	Multivariable models^a^	Crude models	Multivariable models^a^	Crude models	Multivariable models^a^	Crude models	Multivariable models^a^
	
	cOR (95% CI)	aOR (95% CI)	cOR (95% CI)	aOR (95% CI)	cOR (95% CI)	aOR (95% CI)	cOR (95% CI)	aOR (95% CI)
Anticipated HIV stigma	*N* = 305 0.73 (0.60–0.89) *p* = 0.002	*N* = 276 0.79 (0.63–0.98) *p* = 0.034	*N* = 305 0.93 (0.79–1.09) *p* = 0.355	*N* = 276 0.91 (0.76–1.09) *p* = 0.296	*N* = 305 0.86 (0.73–1.03) *p* = 0.103	*N* = 276 0.91 (0.73–1.13) *p* = 0.394	*N* = 284 1.03 (0.87–1.21) *p* = 0.725	*N* = 258 1.03 (0.86–1.23) *p* = 0.753
Self-efficacy	*N* = 305 0.97 (0.89–1.06) *p* = 0.504	*N* = 276 1.04 (0.93–1.16) *p* = 0.547	*N* = 305 1.00 (0.95–1.05) *p* = 0.961	*N* = 276 1.00 (0.94–1.05) *p* = 0.925	*N* = 305 0.96 (0.90–1.02) *p* = 0.204	*N* = 276 1.01 (0.92–1.10) *p* = 0.911	*N* = 284 1.02 (0.97–1.07) *p* = 0.567	*N* = 258 1.01 (0.95–1.07) *p* = 0.814
Fatalism	*N* = 305 0.98 (0.96–1.00) *p* = 0.060	*N* = 276 0.99 (0.96–1.01) *p* = 0.224	*N* = 305 1.00 (0.99–1.01) *p* = 0.755	*N* = 276 1.00 (0.98–1.01) *p* = 0.440	*N* = 305 1.00 (0.99–1.01) *p* = 0.972	*N* = 276 1.02 (1.00–1.04) *p* = 0.138	*N* = 284 1.00 (0.99–1.01) *p* = 0.546	*N* = 258 1.00 (0.99–1.02) *p* = 0.919
Gender equity	*N* = 305 1.00 (0.95–1.05) *p* = 0.972	*N* = 276 1.01 (0.96–1.07) *p* = 0.664	*N* = 305 1.02 (0.99–1.05) *p* = 0.159	*N* = 276 1.01 (0.98–1.04) *p* = 0.439	*N* = 305 0.96 (0.93–1.00) *p* = 0.032	*N* = 276 0.97 (0.93–1.10) *p* = 0.128	*N* = 284 1.00 (0.98–1.03) *p* = 0.994	*N* = 258 0.98 (0.95–1.01) *p* = 0.204
Sensation-seeking	*N* = 305 1.08 (0.90–1.30) *p* = 0.403	*N* = 276 0.96 (0.77–1.19) *p* = 0.681	*N* = 305 0.99 (0.91–1.08) *p* = 0.856	*N* = 276 0.99 (0.89–1.10) *p* = 0.876	*N* = 305 1.14 (1.00–1.30) *p* = 0.049	*N* = 276 1.05 (0.89–1.24) *p* = 0.536	*N* = 284 0.95, 0.86–1.04) *p* = 0.249	*N* = 258 0.94 (0.84–1.06) *p* = 0.318
Self-esteem	*N* = 284 1.11 (1.01–1.23) *p* = 0.029	*N* = 258 1.06 (0.94–1.20) *p* = 0.344	*N* = 305 0.98 (0.94–1.02) *p* = 0.286	*N* = 258 1.01 (0.96–1.06) *p* = 0.808	*N* = 284 1.02 (0.97–1.08) *p* = 0.430	*N* = 258 0.94 (0.87–1.01) *p* = 0.107	*N* = 284 1.03 (0.98–1.07) *p* = 0.226	*N* = 258 1.06 (1.00–1.12) *p* = 0.038

a All multivariable models were adjusted for age, religion, educational level, marital status, monthly income, years worked as truck driver, and clinic. In addition, the models for accepting testing at baseline and testing at follow-up were adjusted for randomisation assignment.

aOR = adjusted odds ratio; CI = confidence interval; cOR = crude odds ratio
